# Liquid Metal Antennas: Materials, Fabrication and Applications

**DOI:** 10.3390/s20010177

**Published:** 2019-12-28

**Authors:** Kashif Nisar Paracha, Arslan Dawood Butt, Ali S. Alghamdi, Suleiman Aliyu Babale, Ping Jack Soh

**Affiliations:** 1 Department of Electrical Engineering, Government College University Faisalabad, Faisalabad 38000, Pakistan; kashifnisar@gcuf.edu.pk; 2 Department of Electrical Engineering, College of Engineering, Majmaah University, Majmaah 11952, Saudi Arabia; 3 Department of Electrical Engineering, Bayero University Kano, Kano 700241, Nigeria; sababale.ele@buk.edu.ng; 4 Advanced Communication Engineering (ACE) Centre of Excellence, School of Computer and Communication Engineering, Universiti Malaysia Perlis, Perlis 02600, Malaysia; pjsoh@unimap.edu.my

**Keywords:** flexible antenna, liquid metal, wearable antenna, fluidic antenna, reconfigurable antenna, antenna array, microfluidic channels, patterning techniques, EGaIn, Galinstan

## Abstract

This work reviews design aspects of liquid metal antennas and their corresponding applications. In the age of modern wireless communication technologies, adaptability and versatility have become highly attractive features of any communication device. Compared to traditional conductors like copper, the flow property and lack of elasticity limit of conductive fluids, makes them an ideal alternative for applications demanding mechanically flexible antennas. These fluidic properties also allow innovative antenna fabrication techniques like 3D printing, injecting, or spraying the conductive fluid on rigid/flexible substrates. Such fluids can also be easily manipulated to implement reconfigurability in liquid antennas using methods like micro pumping or electrochemically controlled capillary action as compared to traditional approaches like high-frequency switching. In this work, we discuss attributes of widely used conductive fluids, their novel patterning/fabrication techniques, and their corresponding state-of-the-art applications.

## 1. Introduction

To fulfill the needs of modern wireless communication technologies, reconfigurable antennas, with their adaptability and diversified functionality, have become an increasingly attractive feature in future communication devices. The reconfigurability feature in antennas is generally implemented using high-frequency switching and other similar techniques to vary their frequency of operation, its pattern, and polarization without integrating multiple antennas within the same device. Conventional reconfigurable antennas are generally developed using highly conductive metals such as copper, which results in highly efficient antennas, but lacks mechanical flexibility. Such limitation reduces the application range of copper-based antennas to rigid or slightly conformal host surfaces, depending on their thicknesses. Fluidic antennas, on the other hand, adopt the mechanical properties of the encasing material. The liquid conductors contained in fluidic channels allows it to take the shape of these channels typically enclosed in a substrate due to its low viscosity [1]. Hence, fluidic antennas can withstand mechanical deformation such as bending, stretching, folding, and twisting when used in flexible substrates, and are able to return to their original shape due to its highly reversible nature [2].

Most of the conventional antennas are fabricated by etching the copper cladding on the rigid substrates to form static conductor shapes. Such antennas are highly efficient but suffer from irreversible structure deformation and even damage when being bent or stretched beyond certain limits [3]. As alternatives, several types of copper-coated polymer substrates such as Kapton, polyethylene terephthalate (PET), and polyethylene naphthalate (PEN) films have been investigated to accommodate the need in flexible applications such as antennas. Similar to conventional substrates, the electrical properties of these substrates also degrade after severe bending/stretching, which in turn reduces antenna efficiency. Another option is polymer-based materials such as polydimethylsiloxane (PDMS), which has been typically used in combination with copper foils to realize highly flexible antennas. However, the semi-rigid copper foils may potentially suffer from irreversible deformation after certain bending cycles. Alternatively, liquid metals can be injected into microfluidic channels to fabricate highly flexible and mechanically stable antennas without compromising their electrical properties [4]. 

In [5], a broader review of fluidics-based tuning methods for the development of microwave components was presented. Although, this included a review of antennas based on dielectric/conductive fluids, it primarily covered flexible and frequency-tunable antenna/arrays and lacked depth on polarization/pattern reconfigurability techniques. Additionally, discussion of recent advancements in state-of-the-art liquid metal applications was also missing in [5]. In [6], a mini review of flexible and stretchable antennas based on textile materials was discussed. Here, the focus was on the benefits of conductive filler-based elastomers used in antennas for bio-integrated electronics applications and the trade-off between antenna stretchability and its performance. However, a detailed review of materials, novel fabrication techniques, and diverse applications was not discussed in that work. In another work, liquid metals (LMs) and general techniques for fabrication of flexible electronics using liquid metals were discussed [7]. This work focused on various LM patterning and encapsulation techniques, but lacked depth on antenna applications.

This review paper focuses on the design aspects of materials and fabrication techniques used in development of state-of-the-art liquid metal antennas alongside their applications. In Section 2 of this work, a review of widely used materials for development of radiating elements and substrates for LM antennas is performed. Later in Section 3, various fabrication techniques available in literature for realization of flexible and stretchable LM patterns alongside the corresponding antenna structures are discussed to identify the adopted design strategies. The wide range of methods/techniques adopted in LM antennas to achieve reconfigurability are also summarized in Section 4. Finally, developed LM antennas for various wearable, implantable, cellular device, and array applications are discussed in Section 5 before reaching conclusive remarks in Section 6.

## 2. Materials in Liquid Metal Antennas

In antenna design, the available fluidic antenna materials can generally be classified into non-conductive, partially conductive, and conductive fluids. Non-conductive fluids include de-ionized (DI) water [8,9], ethyl acetate [10,11], acetone [12], and various oils such as mineral/transformer oil [13,14], which are used in dielectric resonator antennas (DRA). Partially conductive fluids like seawater [15,16] are also available and typically used in fabricating antennas for maritime applications, while solute concentrations in electrolytic solutions [17] can be used to fine-tune antenna performance. Finally, the third type, which this review focuses primarily on, is conductive fluids. Liquid conductive metals because of their solid-like oxide skin on the surface, impart mechanical stability to the elastomeric antennas and hence, provide flexibility while maintaining high conductivity suitable for antenna applications.

Generally, solid conductors such as copper exhibit excellent properties as an antenna’s conductive element. However, their rigid nature limits their use for applications requiring flexibility. To overcome this, thin metallic films have been used for fabricating flexible electronics. However, such flexibility has its limits, as the excessive stretching/twisting will eventually result in micro cracks in these conductors, thus increasing risks of breakdown in flexible antenna applications. Embedded antennas within elastomers such as PDMS etc., may use thin metallic wires, which are capable of providing elasticity and satisfactory conductivity [18]. However, unnecessary design constraints (limitation of stencil dimensions used in patterning of PDMS + silver nanowire (AgNW) composite) along with fabrication complexity also make this approach unsuitable for low-cost flexible antennas. 

**Figure 1:**
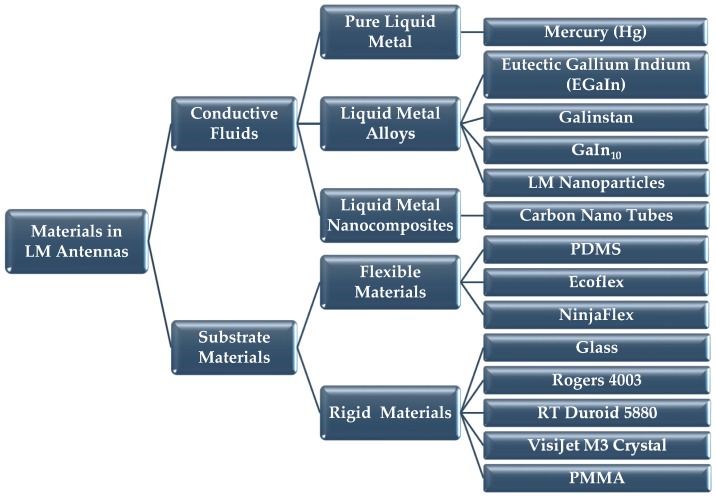
Typical materials used in LM antenna design. Abbreviation: LM, liquid metals; PDMS, polydimethylsiloxane; PMMA, polymethyl methacrylate.

On the other hand, fluidic materials by their very nature have no deformation limits, making them a suitable alternative to rigid or solid conductors for such applications. The lack of a fixed form and the physical properties of conductive fluids have enabled the development of a wide variety of innovative methods and techniques to fabricate LM antennas. Even in cases where the radiative elements are formed on stiff substrates, the liquid nature of used LM materials provide additional degrees of freedom to achieve greater reconfigurability. The most popular approach is to fabricate LM antennas on soft and flexible substrates to realize elastic antenna designs. Such elasticity is not achievable when using solid radiative elements. A summary of the typical conductive fluids and associated substrates are depicted in Figure 1 and discussed below.

### 2.1. Conductive Fluids

Fluids considered conductive enough for use as radiative elements in antennas generally possess electrical conductivities (σ) of the order of 10^6^ S/m as listed in Table 1. These conductive fluids are mostly either based on liquid metals or exist as composites formed by suspended conductive nanostructures/particles in otherwise less conductive fluids. LMs traditionally include the likes of mercury and gallium-based alloys like Galinstan and eutectic gallium indium (EGaIn) [7,19]. However, novel LM based nanocomposites were also developed to further enhance LM’s electrical properties [20].

Compared to solid conductors such as copper, the use of conductive fluid as a radiative element allows for the design of a much more flexible and highly reconfigurable antenna. In particular, Ga alloy-based LMs enjoy rheological properties at room temperature because of the formation of a thin solid-like oxide skin layer which readily forms on the liquid’s surface when exposed to even minute quantities of oxygen in the air [21]. This property allows LM to behave like an elastic material and retain structure till a critical stress is applied on the surface allowing it to flow rapidly. Consequently, this skin oxide layer also provides mechanical stability to the elastomeric antenna after it is filled in a microchannel or is 3D printed [21,22]. This allows an LM fluidic antenna to be highly flexible and deformable while maintaining high conductivity, which is suitable for antenna applications. 

Mercury is the only metal available in liquid form at room temperature with melting point of −39 °C and an electrical conductivity of 1 × 10^6^ S/m [2]. It also has good stiction properties and a low oxidation rate, making it a suitable material for developing a liquid antenna [23]. However, mercury is extremely toxic and must be handled with care, resulting in its limited usage in antenna design.

EGaIn is a liquid metal alloy composed of 75% gallium and 25% indium. It features an electrical conductivity of 3.4 × 10^6^ S/m and a melting point of 16 °C, which is significantly close to room temperature. Most importantly, the non-toxic nature of EGaIn makes it a popular choice for flexible and reconfigurable antennas [4,29,30]. Once exposed to air, this liquid alloy reacts with oxygen to form a thin surface oxide layer, which improves mechanical stability, surface tension, and significantly prevents evaporation. This results in improved performance of EGaIn without impacting the overall conductivity [29].

The commercially available Galinstan is yet another liquid metal alloy widely used as the antenna-radiating element [30,31,32]. It comprises of 68.5% gallium, 21.5% indium and 10% tin with an electrical conductivity similar to EGaIn [33,34]. Galinstan also benefits from the thin surface oxide skin formed when in contact with oxygen, similar to other LM alloys. However, Galinstan has the added advantage of having a melting point much lower than room temperature (−19 °C) and being non-toxic.

GaIn_10_ based LM ink is another conductive fluid composed of 90% gallium, 10% indium and approximately 0.026% oxygen [25]. This conductive fluid possesses an electrical conductivity of 2.9 × 10^6^ S/m, which is slightly less than EGaIn and Galinstan and is formed by vigorously stirring GaIn_10_ alloy in a controlled fashion till enough gallium oxide is formed to make the fluid viscous enough to be used as an LM ink. Similar methods can also be used to prepare conductive inks from other LM materials like Galinstan as reported in [35]. Here, an addition of 0.30% of oxygen into Galinstan by stirring method provides the LM sufficiently enhanced surface tension and adhesion properties for its subsequent use as an LM ink. 

Although electrical conductivity of LMs is around an order of magnitude smaller than copper, this disparity can be reduced by using LM-based composites with suspended conductive nanostructures. This has been achieved for EGaIn, where an addition of 0.5% walled carbon nanotubes (SWNT) resulted in a 100% increase in electrical conductivity of the base fluid [20]. Increased concentration of SWNT resulted in a corresponding increase of electrical conductivity of the nanocomposite, a phenomenon linked to a gradual buildup of interconnected SWNT pathways within the fluid.

Despite major flexibility advantage of LM fluids including long-term stability of their electrical properties, certain LM fluids are known to be corrosive in nature. In particular, Ga in LM alloys might come in contact with the surface of traditional metal interconnects like Cu, Ag, Au, and Al when interfacing with external biasing/output circuitry. The corrosive nature of Ga towards other metals might reduce long-term stability of antenna performance [24,36,37]. However, these issues can be addressed by Ni-plating the LM/metal pin interface or by treating it with 1-decylphosphonic acid or hydrochloric acid (HCl) vapor [36]. Additionally, modern composite materials like layers of graphene oxide (GO) together with poly 3,4-ethylenedioxythiophene: polystyrene sulfonate (PEDOT:PSS) can also be utilized [37]. In the composite material PEDOT:PSS/GO, GO blocks the Ga from coming in contact with metal surface while PEDOT:PSS overcomes the nominally insulative nature of GO and ensures good electrical contact between the metal interconnect pin and the LM alloy.

Fluids made out of nanoparticles of highly conductive (but otherwise solid) metals like silver are also available as an alternative to LM for fluidic antenna applications. Similar to [20], the concentration of the nanoparticles modulates the conductivity of such fluids. Silver nanoink [28] is an example of such composite fluids and exhibits far superior electrical conductivity of ∼20 × 10^6^ S/m with 72% suspended Ag nanoparticles by weight. However, such composites are relatively more expensive than traditional LMs.

Both LM and silver-based fluids can be used to develop highly flexible and conductive elastomeric composites. In [18], silver nanowires were embedded in PDMS substrate to achieve an electrical conductivity of 0.813 × 10^6^ S/m. Similarly in [27], suspended EGaIn nanodroplets were formed in ethanol by the sonification method and were later deposited as nanoparticles embedded within the PDMS substrate. Contrary to [18], the elastomer was initially insulative and applied pressure sinters nanoparticles to form interconnected electrical paths. The achieved electrical conductivity in [27] after pressure sintering was ∼0.1 × 10^6^ S/m, which is an order of magnitude smaller than standard LM fluids. 

### 2.2. Substrate Materials

Substrates in planar antennas provide the much-needed mechanical support to the radiating element. For the case of LM antennas, the LM is either encapsulated by the substrate or being flowed/sprayed onto the surface of the designed substrate. Substrate materials can be broadly grouped into two categories based on their flexibility, as depicted in Figure 1. In [38], Rogers/RT Duroid 5880 rigid material has been used as an antenna substrate together with a VeroClear based 3D-printed microfluidic encapsulation for the liquid metal. Here, RT Duroid 5880 is a printed circuit board (PCB)-like commercially available high-frequency laminate formed by polytetrafluoroethylene (PTFE) composites reinforced with glass microfibers while VeroClear is a commercially available acrylic-based rigid material used for 3D printing of optically clear structures. Another 3D-printed rigid substrate material is VisiJet M3 Crystal which was used in [39] to develop a planar patch and helical antenna using a vacuum filling technique. Meanwhile in [9], Rogers 4003C rigid substrate was milled before a second top cover made of the same material was glued to the first one, forming a sealed fluidic channel. Here, Rogers 4003C is a commercially available material composed of hydrocarbon/ceramics with reinforced woven glass, typically used for high frequency PCB design. Next, a low cost rigid LM patch antenna was developed in [40] using Rogers Duroid 5880 substrate and a polyamide mask. In [41], a polymethyl methacrylate (PMMA) based rigid substrate material was used to develop a reconfigurable monopole antenna. Here, the fluidic channel surface was coated with a commercially available non-stick coating of “NeverWet” which stops wetting of LM solid oxide to the substrate and allows reversible actuation of LM inside the closed channel. 

Among flexible materials, a commercial elastomer known as PDMS [42] is the most common material used for developing microfluidic channels, which is to be consequently injected by LM to form antennas [4,23,29]. PDMS, with a dielectric constant of 2.67, is not only highly elastic but also possesses a low surface energy and Young’s modulus. PDMS is also used as an encapsulation layer in antennas after the antenna has been designed using other methods [20,43,44]. Several other commercially available flexible materials include EcoFlex silicone rubber [45] and thermoplastic polyurethane (TPU) based NinjaFlex [31], which are generally 3D printed to have desired LM patterning features. 

## 3. Patterning Methods for Liquid Antenna Fabrication

Conventional antennas are generally fabricated by the etching of the copper cladded rigid substrate with static shapes. As aforementioned in Section 2, copper-based antennas are highly efficient but suffer from irreversible structure deformation when bent or stretched beyond certain mechanical limits [3]. Limited flexibility is achieved by fabricating thin layers of solid metals on elastomeric substrates. However, even these approaches suffer from irreversible deformation after certain bending cycles [1]. Alternatively, the liquid metals patterning methods are actively being researched for applications like stretchable and soft electronics [22,46,47]. A wide variety of LM patterning techniques for fabrication of general electronic circuits including microwave components were discussed in [48]. Most of the same fabrication/patterning techniques for conventional antennas, including the soft lithographic method [4], direct wiring technique [49], stencil printing, 3D printing [50], and substrate milling [9], also apply to LM antennas. In addition to that, most LM antennas are fabricated using a multitude of methods simultaneously. Nonetheless, based on its distinct features, LM antenna fabrication techniques can be broadly categorized into three main types, which are soft/photo-lithography, 2D/3D printing, and LM spraying onto the substrate.

### 3.1. Soft/Photo-Lithography

Among the LM patterning techniques, lithography is the most widely used method for developing rigid molds for flexible prototype antenna development. These molds are primarily used for flexible substrates to create microfluidic channels to be filled with LM fluid. This process was used in [4] where a mold, developed using photolithographic process on silicon wafer, was used to create replica PDMS molds of a dipole antenna. The developed 150 μm thick microfluidic channels in the PDMS substrates were later filled with EGaIn via injection.

**Figure 2:**
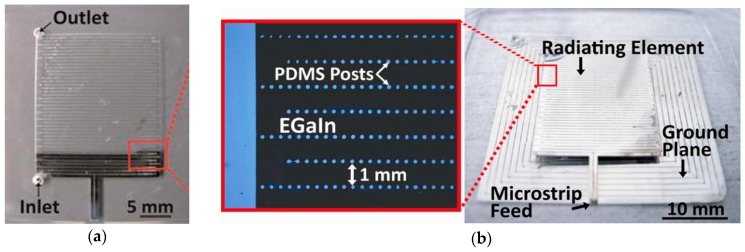

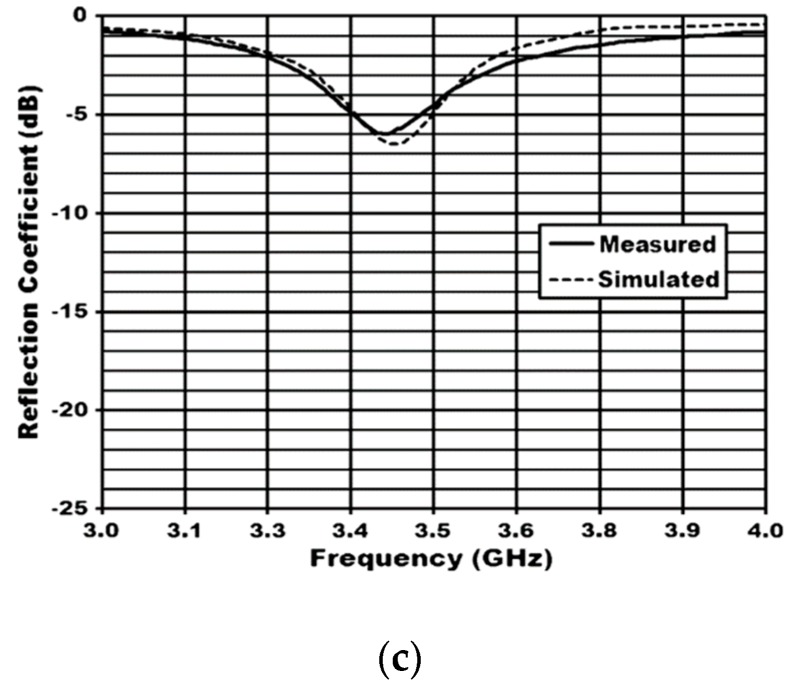
Microstrip patch antenna: (**a**) partially; (**b**) fully filled with EGaIn, inset on left depicting PDMS posts to ensure even liquid filling; and (**c**) simulated and measured response of the microstrip antenna [29].

Similarly, a multi-layered microstrip patch antenna with two PDMS layers and microchannels fabricated using soft lithography is depicted in Figure 2 [29]. These conductive layers acted as the ground plane and radiating element, and consisted of micro PDMS structures. These structures ensured the even filling of the injected EGaIn fluid. Measurements of this flexible antenna showed good conformity between the simulated and measured response at 3.45 GHz, as shown in Figure 2c. From Figure 2, it is evident that a complex structure can be realized using soft-lithographic process and LM. In [43], Galinstan channels were created between two PDMS layers using a series of processes including photo-lithography. This was aimed at forming a radio-frequency (RF) coil antenna to transfer power and data while being flexible and elastic in nature.

**Figure 3:**
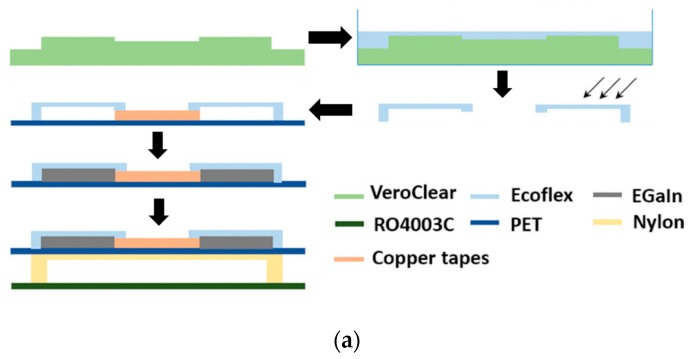

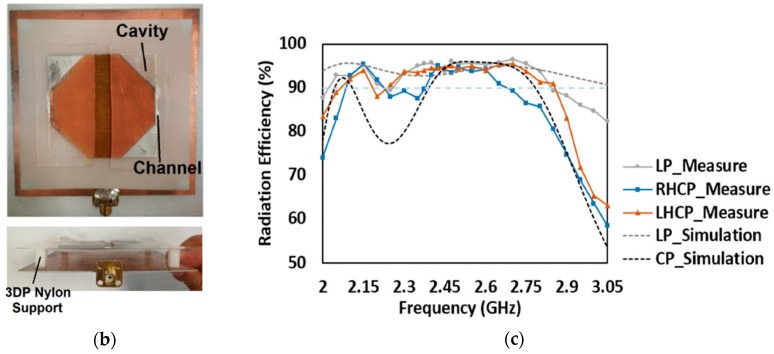
(**a**) Fabrication process of the patch antenna using soft-lithography. (**b**) Front and top view of the fabricated antennas. (**c**) Comparison of antenna’s simulated and measured radiation efficiency [45]. Abbreviations: PET, polyethylene terephthalate.

Several other non-flexible antennas also utilize soft-lithographic methods to generate micro-channels. This is generally achieved by generating micro-channels in the elastomeric substrates to be consequently attached to standard PCBs using some adhesives. However, the adhesive layer must be removed from under the microchannels to allow higher uniformity in the LM throughout the channel. This process was reported in [23], where a frequency reconfigurable monopole antenna was developed using PDMS, liquid crystal polymer (LCP) and RT 5880 substrates with mercury as the liquid metal. Soft lithography method was used to develop a microfluidic channel in the PDMS substrate, and the radiating LM’s length was manipulated using a micro pump to reconfigure the antenna’s frequency of operation. Similarly, a VeroClear 3D mold was used in [45] to generate a top LM encapsulation layer made of Ecoflex material. This layer was fixed on a PET sheet using copper tapes as depicted in Figure 3a. EGaIn was later injected into the gap and the entire assembly was fixed onto a Rogers 4003C substrate using a nylon stand to form a patch antenna (see Figure 3b) with good conformity between simulated and measured radiation efficiency performance, as shown in Figure 3c.

In [27], a dipole antenna was fabricated using lithography replica molding to generate PDMS microchannels. The fluid used in this work was a mixture of suspended EGaIn nanoparticles in ethanol. Here, the mixture was first injected into the microchannels where ethanol naturally evaporated due to its gas permeability and left behind LM nanoparticles. These LM nanoparticles were insulative owing to encapsulation by a skin oxide layer but underwent sintering once mechanical pressure was applied onto the PDMS encapsulation to generate conductive pathways. Compared to pure EGaIn, nanoparticle mixture reduced the pressure needed to fill a 50 µm × 300 µm microchannel from 45 kPa to 15 kPa. This reduction in filling pressure was achieved as the nanoparticles suspended in ethanol primarily exhibited the flow properties of the solvent instead of pure EGaIn, which has a larger surface tension. 

Instead of simply injecting LM into patterned microchannels, the conductive fluid can also be introduced into the channels using the vacuum filling technique, as reported in [51]. Here the microchannel structure, fabricated using soft lithography on PDMS substrate was placed in a vacuum chamber to evacuate air from the channels. Later, once the atmospheric pressure was restored, the EGaIn LM drop (placed on the microchannel inlet prior to vacuum creation) was pulled into the microchannel. This technique was used to fill highly complex and dense microchannels with relative ease, including a coil antenna with ∼60 cm of microchannel length, as reported in [51]. Compared to the standard injection method, this technique has multiple advantages as it allows complete filling of dead ends without any outlet and can allow fabrication of LM antennas with microchannel thickness as small as a few microns. A summary of the discussed antenna fabrication techniques using soft/photo-lithographic method, including both injection and vacuum filling method, is detailed in Table 2.

### 3.2. Printing

In addition to soft lithographic methods, liquid metal can also be filled in the channels/cavities of 3D-printed flexible substrates as detailed in [22]. 3D printing is a kind of additive method where the antenna’s complex housing structure (microfluidic channels) is printed onto the target substrate mostly using the FDM (fused deposition modelling) method. In this method, plastics such as NinjaFlex are melted to print complex antenna structures. 

**Figure 4:**
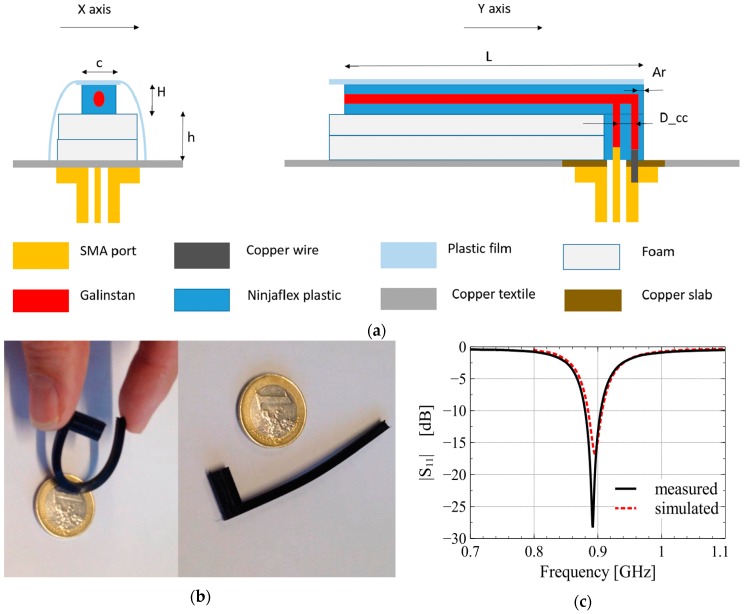
(**a**) Structure of miniaturized inverted F antenna using 3D-printed NinjaFlex plastic; (**b**) fabricated antenna depicting structural flexibility; (**c**) measured versus simulated antenna reflection coefficients [31].

In [31], Galinstan and 3D-printed NinjaFlex substrate were used to fabricate an inverted-F antenna (IFA). The fluidic channels present within the substrate were injected with Galinstan to form the radiating element, as shown in Figure 4. The antenna fabricated using this method was highly flexible, as depicted in Figure 4b. Similarly, microfluidic channels for EGaIn were 3D printed using VeroClear substrate to form a patch antenna with switchable slots (PASS) [38]. Meanwhile, in [39], Visijet M3 Crystal, a commercially available photocurable resin, was 3D printed to form planar and helical antennas with EGaIn as the radiative element. Once the substrate was 3D printed and the supporting structure was properly dissolved, LM was introduced into its cavities using a vacuum filling technique. Antennas fabricated using this method were promising only till 8 GHz frequency, because higher frequency designs required printing accuracy and feature sizes currently unattainable with the used technology. Similarly in [52], the same substrate, LM fluid, and filling technique was used to develop a more complicated LM array having complex support structures.

The 3D-printed substrates alongside LM can also be incorporated in dielectric resonant antennas (DRAs), as depicted in [53]. Here, polarization reconfigurability was introduced by inserting a 3D-printed cylindrical layer between two glass DRA layers above a PCB. The printed layer was made of a high-temperature resin with two orthogonal cavities. Introduction of Galinstan in any of the cavities, changed the polarization of the DRA.

**Figure 5:**
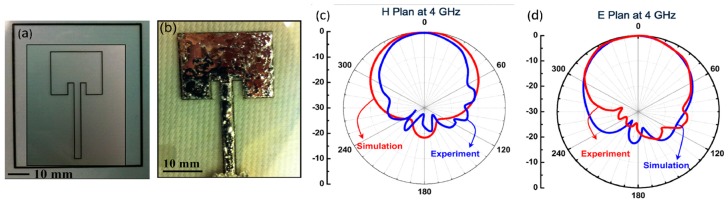
(**a**) Antenna frame generated by UV-assisted direct writing technology on a PDMS substrate; (**b**) fabricated antenna with EGaIn/SWNTs nanocomposite; (**c**) H-plane; and (**d**) E-plane pattern comparison for simulated and measured response [20].

As an alternative to 3D printing channels and cavities, direct writing of antenna structure or printing of conductive traces is also possible. Figure 5 depicts one such method where UV-assisted direct write technology was used to design antenna frame on a PDMS substrate [20]. To increase the mechanical durability and electrical conductivity of EGaIn, single-walled carbon nanotubes were incorporated to fabricate this fluidic patch antenna operating at 4 GHz (S-band). A 100% increase was observed in terms of electrical conductivity of the LM when using this approach, relative to the use of pure EGaIn metal alloy. Furthermore in [26], frontend inductive coils of radio-frequency identification (RFID) tags made of GaIn_10_ LM ink [25] were direct printed on various flexible substrates like paper, plastic, latex, and textile materials using a novel LM ink printer. With a 200 µm thick conductive trace and accuracy of up to a micron, this low-cost direct printing method showed a promising prospect for future fluidic antenna design and fabrication.

**Figure 6:**
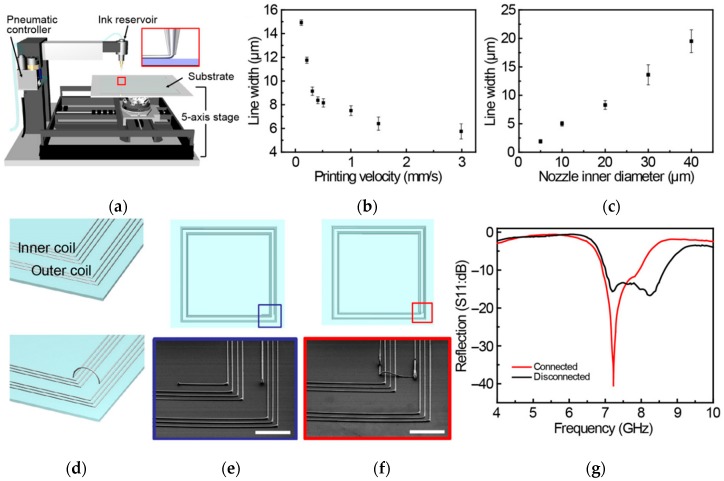
(**a**) Developed 3D printer; (**b**) relation between 3D-printed EGaIn linewidth and printing speed; (**c**) relation between printer’s nozzle diameter and printed line width; (**d**) schematic of reconfigurable RF antenna coil; (**e**) schematic and SEM image of fabricated antenna with disconnected and (**f**) connected inner/outer coils (scale bar was 300 µm); and (**g**) corresponding variation in reflection co-efficient of reconfigurable antenna in both states [54].

In [54], there was a novel high-resolution printer for 3D direct printing of LM alloys with track thickness as small as ∼2 µm, as depicted in Figure 6 [54]. Here, the rapid oxidation property of LM alloys like EGaIn together with nozzle lift velocity manipulation allowed 3D printing of the conductive tracks. To demonstrate this property, a pair of nested disconnected RF coils were first developed on glass substrate by 2D printing LM tracks with width/spacing of 12 µm/50 µm. The reconfigurability was later achieved by connecting the two coils using 3D direct printing, as depicted in Figure 6d–f. This novel printer has demonstrated printing of ultra-thin conductive LM tracks in 3D with high reliability and repeatability, and does represent the state-of-the-art among LM antenna fabrication techniques. A summary of the discussed antenna fabrication techniques using 3D-printed substrates, support structures or by using direct writing/printing of LM inks is detailed in Table 3.

### 3.3. Spraying

**Figure 7:**
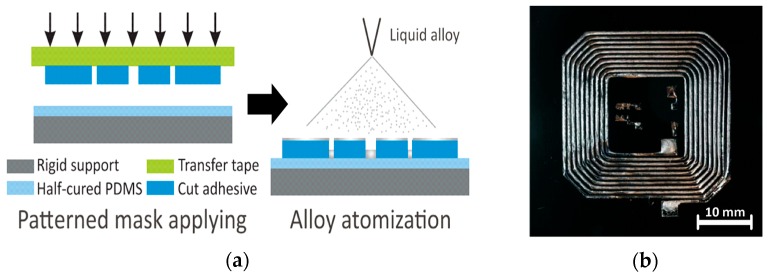

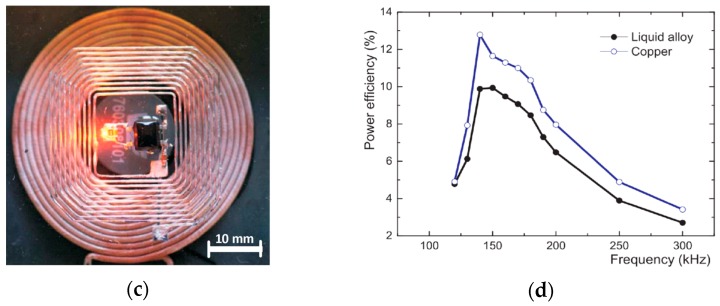
(**a**) Schematic illustrating resonant coil antenna fabrication using PDMS patterning and LM atomization spray; (**b**) fabricated resonant coil filled with liquid alloy; (**c**) resonant coil in operation; and (**d**) comparison of power transfer efficiency of resonant coil antenna with a standard copper coil [44].

A liquid metal can be sprayed to form conductive traces on the elastomeric substrate. This method can be seen implemented in Figure 7a, where a transfer tape with attached cut adhesive was used to transfer the pattern outline to a half cured PDMS layer [44]. Later, the transferred adhesive layer was subjected to atomized LM spray. This was to form a power transfer coil with traces of up to 200 µm thickness as shown in Figure 7b,c. A comparison of the proposed RF coil antenna to a similar copper coil is presented in Figure 7d and showed good prospects for the RF coil, considering the added advantage of flexibility. Similarly, Galinstan was sprayed using a pressurized air gun to form pattern of patch antenna on a Rogers 5880 substrate in [40]. The performance of the LM patch antenna was comparable with its copper counterpart, which reached up to 98% of radiation efficiency with maximum gain of 5.26 dBi.

LM patterning can also be achieved by pressure spraying atomized microdroplets of conductive fluids onto a screen covering the substrate, as reported in [35]. In this particular work, Galinstan LM ink patterns were generated by spraying the LM on to substrates like paper, PVC, and PDMS using polyester screens. These screens have meshed openings as small as 75 µm each to allow LM microdroplets to seep through, while the emulsion covering on remaining screen blocks such seepage. This technique allows quick patterning of track widths as small as ∼250 µm and was used to develop an RFID antenna on PVC material in [35]. The discussed antenna fabrication techniques using LM spraying method are listed in Table 4.

## 4. Reconfigurability in LM Antennas

Due to the integration of various wireless technologies/standards in the future 5G network, the requirement for reconfigurable antenna with frequency/polarization tunability is highly desirable. Traditionally, they are implemented using different types of surface-mounted switches such as positive-intrinsic-negative (PIN) diodes, field effect transistor (FET), inductors, and varactor diodes [55,56,57]. These methods are not only power consuming with less tunability range, but also cause nonlinearity in terms of undesirable frequency components. Alternatively, frequency agility can be achieved by employing ferromagnetic or liquid crystal materials [58]. Though this method has low power consumption losses, it suffers from a complex tuning process alongside limited frequency tunability.

Recently, fluidic antennas were widely used to implement reconfigurability by changing the physical size of the antenna. The fluidic-based methods have three main advantages: (a) they are beneficial in high power RF application because of their power handling capabilities; (b) they do not require extra biasing circuitry for tunabilty, as in the case of conventional tuning components, and thus can be designed to be less complex; and (c) flexible and wearable antenna compatible with flexible electronics can be realized due to soft material being used in fluidic antenna. It is worth mentioning that the RF power handling capabilities of reconfigurable LM antennas was highly emphasized in literature without a proper discussion on its capabilities and limitations. Nonetheless in [59], a 5 W @2.5 GHz limit was associated to a Hg based monopole antenna with PDMS substrate [60]. This limit was instilled primarily due to the temperature limitation (70 °C) of the micropumps used to achieve reconfigurability. Other main limitations of the LM-based reconfigurability techniques were the low tuning speed and the size of the micropump.

Although reconfigurability, in general, can be achieved in a wide range of LM antenna parameters including its gain as reported in [61] for an electrically actuated patch antenna, this section focuses primarily on the frequency, polarization, and pattern reconfigurability aspects of the LM antennas as discussed below.

### 4.1. Frequency Reconfigurable Antennas

**Figure 8:**
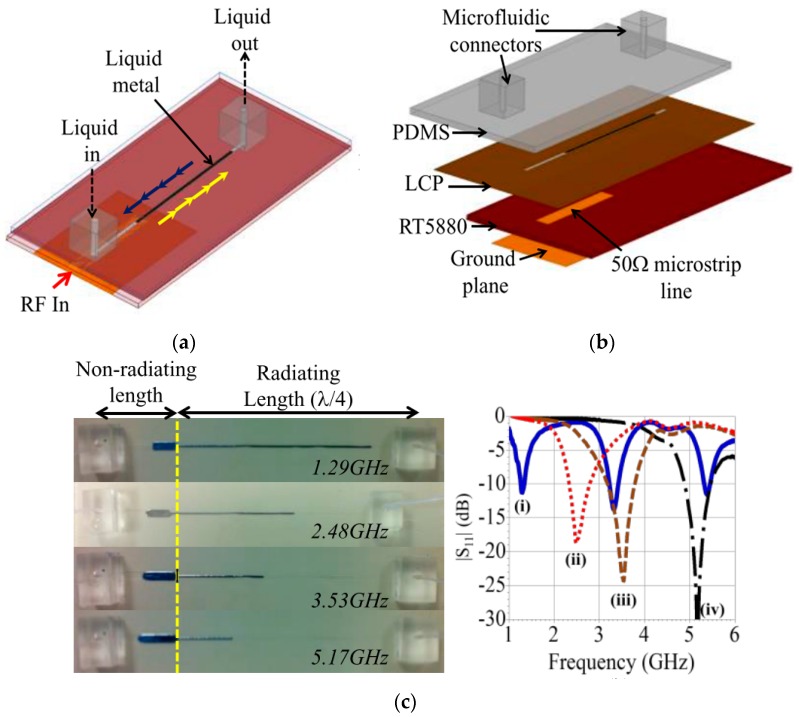
(**a**) Design of frequency reconfigurable monopole fluidic antenna with mercury as the fluid; (**b**) exploded view of antenna layers; (**c**) fabricated antenna and corresponding frequency response [23].

**Figure 9:**
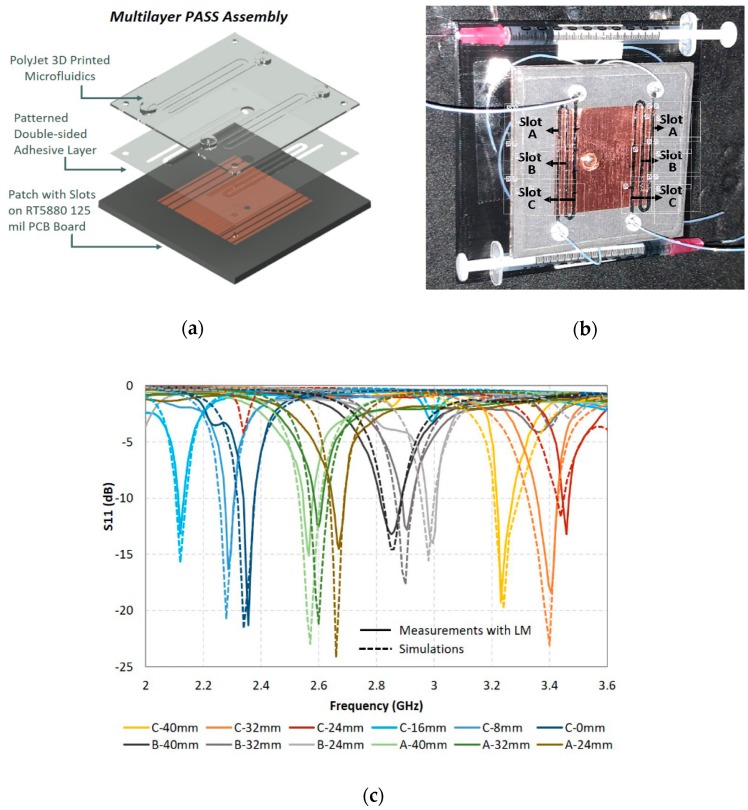
(**a**) Exploded view of the prototype PASS antenna design with VeroClear 3D-printed microfluidics; (**b**) fabricated PASS antenna with labelled slots filled with EGaIn carried by mineral oil; (**c**) PASS S11 parameters as a function of frequency measured for various slot lengths [38].

Frequency reconfigurability can be achieved by using the flow property of conductive fluids if the amount of fluid in cavities can be controlled using micro pumps. This was achieved in [23] where a wideband frequency-reconfigurable monopole antenna using mercury was proposed (see Figure 8). Here, the low oxidization and stiction properties of mercury allowed easy control of the amount of LM in the channel. Figure 8c depicts how movement of mercury had tuned the resonant frequency from 1.29 GHz to 5.17 GHz. Similarly, the flow of the EGaIn could be manipulated by flowing it through a meander line forming multiple slots. This was aimed to tune the antenna frequency from 2.12 to 3.46 GHz, as depicted in Figure 9 [38].

The same frequency tuning can also be achieved using electrochemically controlled capillary action, as proposed in [62]. Here, EGaIn was immersed in NaOH carrier fluid, which stops the formation of the oxide stiction around the LM plug under nominal condition. However, appropriately applied electrical potential difference, the oxide layer was generated within the channel to halt LM movement. The electrochemical capillary action allowed for a 0.66 GHz to 3.4 GHz once the LM length was varied from 75 mm to 4 mm within the channel. Additionally, in flexible LM antennas, the deformation of the antenna shape also impacted antenna frequency and can be used as a stress or strain sensor [43].

### 4.2. Polarization Reconfigurable Antennas

Periodic surfaces such as metamaterials are being used to change the polarization of the incident EM waves from the antenna. To make them reconfigurable, switching components are used at the cost of complexity in design with increased insertion losses [63]. In [64], polarization reconfigurability was achieved by injecting micro fluids (EGaIn) into separate inlets on the MetaSurface (MS). The empty microfluidic channels caused the antenna to operate in the linear polarization (LP) state. Meanwhile, upon the filling up of the upper and lower channels, the polarization was converted to left-hand circular polarization (LHCP) and right-hand circular polarization (RHCP), respectively. This technique was beneficial for the MS using physical switches to enable tunability for antenna polarization [65]. However, proper surface treatment was needed to avoid stiction of the solid oxide layer to the surface of the microfluidic channel, thus making the required task challenging. 

**Figure 10:**
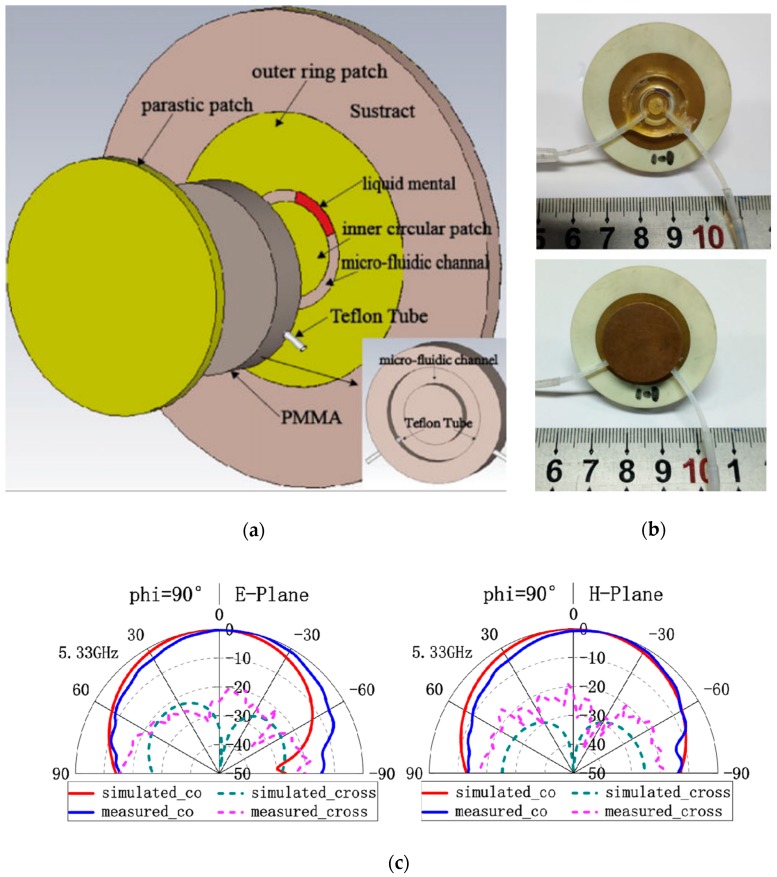
(**a**) 3D exploded design of the circular patch antenna depicting LM in PMMA microfluidic channel; (**b**) Fabricated antenna with and without covering of microfluidic channel; and (**c**) Measured and simulated co/cross-polarization patterns at 5.33 GHz [66].

An antenna with continuously tunable linear polarization from 0° to 180° was achieved in [66] using a parasitic circular patch antenna, as depicted in Figure 10. Here, a circular microchannel fabricated in PMMA cylinder was fixed between two circular patches. The microchannel was filled with a small quantity of EGaIn and the polarization could be tuned by moving LM within the channel.

Polarization reconfigurability could also be introduced into antennas by fabricating multiple LM channels or cavities. This individually or collectively may result in different polarizations. Later on, LM can be inserted or removed from these channels to switch polarizations. Such an approach was used in [53], where a dielectric antenna resonator was developed. Here, the movement of the LM among channels switches antenna polarization between the *y*-axis, +45° and −45° states. The same approach was used in [45], where four corners of a patch antenna were truncated and replaced with cavities for LM insertion. Only two cavities were filled at a time resulting in LP, LHCP, and RHCP polarization states.

### 4.3. Pattern Reconfigurable Antennas

In [67], a liquid filled reflector was used to change the beamwidth of the impulse radiating antenna (IRA). This was done by changing the effective length of the reflector for effective scanning in radar applications. Galinstan was injected/withdrawn in the 34 liquid channels made of Teflon substrate, which were supported by acrylonitrile butadiene styrene copolymer (ABS, ε_r_ ≈ 2.9) structure. The achieved variation of the 3 dB beamwidth ranged from 50.8° at 1.0 GHz to 2.7° at 5.0 GHz. 

**Figure 11:**
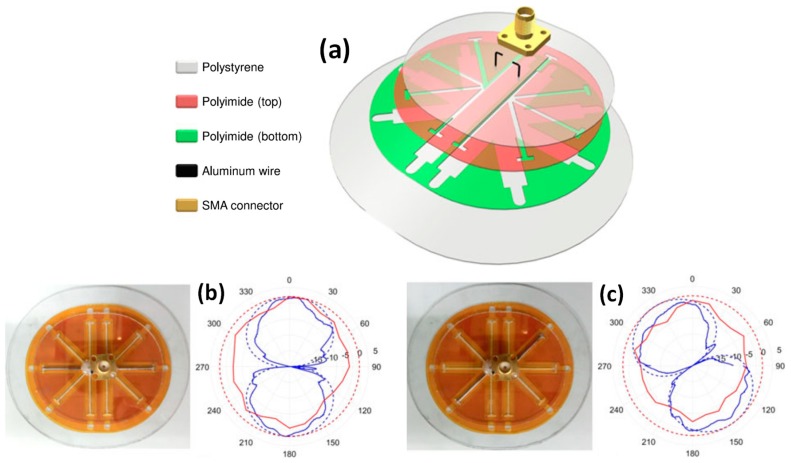
(**a**) Exploded view of the polarization/pattern reconfigurable dipole antenna; (**b**,**c**) represent fabricated antenna with different arms filled with Galinstan to reconfigure pattern with the corresponding simulated (solid) and measured (dashed) of E- (blue) and H- plane (red) results shown on the right [32].

Similar to frequency reconfigurability, electrochemical capillary action can also be used to achieve pattern and polarization reconfigurability as well. Both possibilities were demonstrated simultaneously in the dipole antenna depicted in Figure 11 [32]. Here, each dipole element can be reconfigured into one of the five available arms. The fabricated antenna with two reconfiguration states and corresponding pattern are depicted in Figure 11b,c.

In [68], a pattern reconfigurable antenna was demonstrated at 1.8 GHz frequency with beam steering capability over a 360° range. Here, fine-tuned beam steering could be achieved by altering position of liquid metal in parasitic elements around a novel circular Yagi-Uda antenna. The position of metal liquid (mercury) was controlled by piezoelectric micro pump. The main advantage of replacing solid wire parasitics with LM in such antennas is that continuous tuning of antenna pattern can be achieved by physical displacement of fluid using techniques like pumping or electrowetting.

## 5. LM Antenna Applications

Efforts in introducing new composite materials to improve LM/substrate performance and fabrication methods have resulted in a wide range of applications that take advantage of the flexibility and flow properties of LM antennas. Although copper-based antennas can withstand moderate bending [69], they undergo plastic deformation beyond 2% strain [4] limiting their use for most applications requiring elastic antennas. On the other hand, LM antennas not only retain their electrical properties despite being severely stretched, bent or twisted, but also exhibit long-term stability with negligible performance degradation over multiple stretching/bending cycles. This makes LM antennas suitable for wearable and implantable applications [70]. Furthermore, the ability of LM antennas to provide a linear response over a wide tuning range without extra biasing circuitry has allowed their use as antenna arrays, electrically small antennas, and recently as a cellular device antenna. These applications along with their class representative examples are discussed in this section.

### 5.1. Wearable LM Antennas

The need for a flexible and stretchable antenna for wearable applications is increasingly urgent with the introduction of 5G and the increased R&D in the internet of things (IOT). Such antennas can be fabricated without LM in the form of metal/polymer composite layers [6] or by weaving fabrics with meshed metal coated fibers [71]. However, the flexibility of LM provides additional options for the development of wearable antennas.

For example, in [72], a microfluidic stretchable planar inverted cone antenna (PICA) operating in the 3–10 GHz frequency band was developed by filling Galinstan into PDMS channels. This extremely low-cost antenna can be stretched, bent, or twisted by up to 40% with only a 30% drop in radiation efficiency in operational frequency band. Similarly in [73], the same materials and technique were used to develop a 900 MHz antenna where the developed integrated device also hosted stiff active electronics on the same PDMS substrate. The RF antenna had a measured drop in radiation efficiency from 94% in unstretched case to 81%, with around 15% elongation. Both antennas can be used for wearable applications including healthcare, fitness, IOT and RFID systems.

In [74], a liquid metal loop antenna for wearable application was introduced. Galinstan was used as its radiating element, whereas flexible polymer was used as its substrate. This antenna operated at 868 MHz with 1.5 mm channel diameter, and this antenna could be tuned to 2.45 GHz (ISM band). Besides that, this antenna was also tested with a variety of conditions such as bending, washing, twisting, and freezing up to −19 °C. Despite vigorous stress tests, it still operated, owing to the use of the flexible and robust material. Moreover, the usage of the flexible material also allowed this antenna to be stretched up to 150% from its original size with acceptable radiation performance.

**Figure 12:**
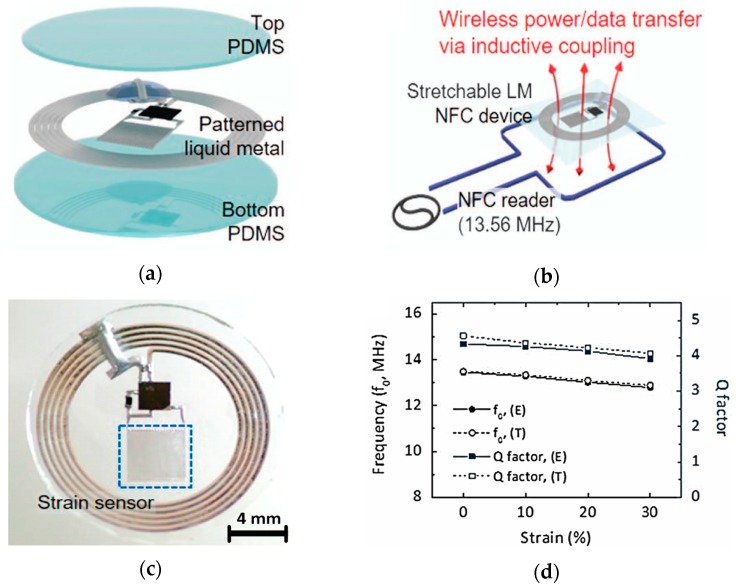
(**a**) Exploded view of the wearable coil antenna with Galinstan LM patterning encapsulated in PDMS encapsulation; (**b**) antenna operation; (**c**) fabricated coil antenna; and (**d**) comparison of experimental and theoretical performance of antenna as a function of strain [43].

In [43], an integrated flexible electronic system was developed, as shown in Figure 12. This skin-attachable motion sensing system consisted of a RF coil antenna for power/data transfer alongside a strain sensor, all fabricated using Galinstan LM. This gel-like electronic sensing system was able to measure strain generated from flexing of skin/muscles based on the variation in resonant frequency, as depicted in Figure 12d. This allowed the monitoring wrist flexion, vocal cord movements, and finger joints. Similarly in [75], a very interesting wearable serpentine microfluidic antenna was developed that mechanically tunes its resonant frequency as a function of applied strain in a predefined direction. The antenna, based on EGaIn fluid in Ecoflex substrate, underwent an almost linear drop in resonant frequency from 1.73 GHz to 1.25 GHz (with radiation efficiency >95%) only once the applied strain on the antenna moves from 0% to 50% in a particular direction. Less than 2% change in resonant frequency was observed for the same mechanical strain applied along a perpendicular direction on the fabricated antenna’s surface. A mutually perpendicular assembly of such antennas on skin surface around muscles or joints can respectively monitor muscle flexing or joint movements.

A wearable LM antenna can also be fabricated within a flexible substrate enclosure such as the 3D-printed NinjaFlex [31]. Simulated and measured reflection coefficients of the Galinstan based IFA indicated good antenna performance at 885 MHz band, with up to 70% efficiency and 3.5 dB gain. Furthermore, bending the 6.65 cm long antenna along the circumference of a hypothetical circular surface (with 5 cm radius) resulted in only a 1% shift in resonant frequency and maximum 10% variation in efficiency.

### 5.2. Compliant LM Antenna Implants

The non-toxic nature of LM alloys like EGaIn encapsulated in biocompatible and stretchable elastomers like PDMS, makes them a suitable choice for developing compliant implantable coils for wireless telemetry applications [76]. Here, the RF coil, developed for subsequent implantation within the human eye, had an elliptical shape and an outer diameter of 16 mm × 10 mm, alongside a fluidic channel cross section area of 200 µm × 640 µm. The developed RF coil for retinal prosthesis had a measured power transfer efficiency of 21% @ 4 MHz with 12 mm of operational distance from the non-LM-based primary coil.

**Figure 13:**
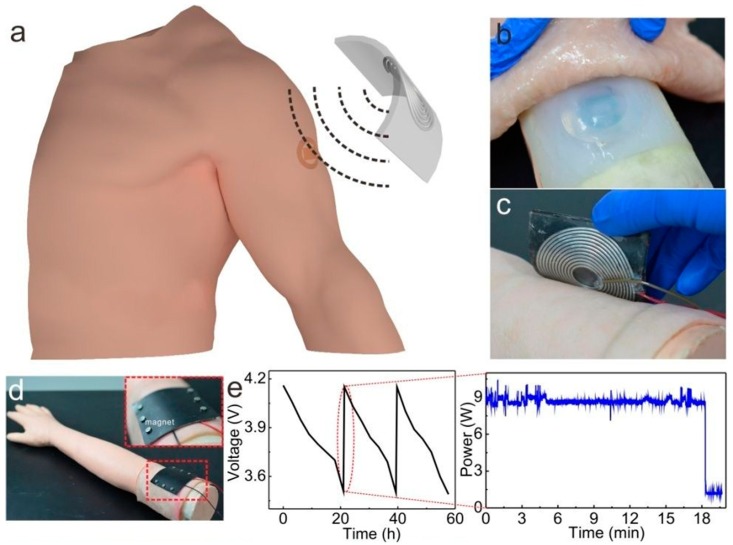
(**a**) Illustration depicting wireless charging of implantable LM coil; (**b**) compliant LM receiver implanted in pigskin; (**c**,**d**) wireless charging of the implanted device; (**e**) change in voltage of the implanted 120 mAh battery during discharge/charge cycles (left) and input RF power at transmission coil during the 18 min charging cycle (right) [77].

In [77], Galinstan based LM coil antennas within PDMS substrates were developed for wireless power transmission applications including wireless charging of implanted devices with battery storage, as depicted in Figure 13. The wireless transmission assembly, tested with a receiver coil under a layer of pig skin to emulate human tissue, was able to fully charge a 120 mAh battery within 18 min with around 9 W of RF power delivered to the transmission coil input. As the involved LM coil was flexible and compliant, it can be incorporated in tissues to ensure a comfortable wireless charging process for patients having biomedical implants with batteries.

### 5.3. LM Antenna for Handheld Cellular Devices

Traditionally, solid copper-based antennas are used in handheld devices. However, recently Galinstan filled in a channel within 32 × 14 × 3 mm^3^ PMMA block was used to develop a reconfigurable multi input multi output (MIMO) antenna for a handheld cellular device [78]. This MIMO consisted of a monopole antenna and a modified inverted F- antenna with a tuning strip and covered eight frequency bands of the long-term evolution advanced (LTE-A) standard. In the developed prototype, a syringe was used to manipulate the movement of LM within the tuning strip. This resulted in the change of the IFA’s length and correspondingly altered the antenna’s operational frequency. Although MIMO antennas were not used in handsets below 1 GHz to avoid mutual coupling between the constituent antennas, the developed prototype showed good isolation properties. This was primarily due to an L-shaped conductive strip placed on the back side of the device which also provided up to 10 dB of additional isolation between the two antennas. 

### 5.4. Electrically Small LM Antenna

**Figure 14:**
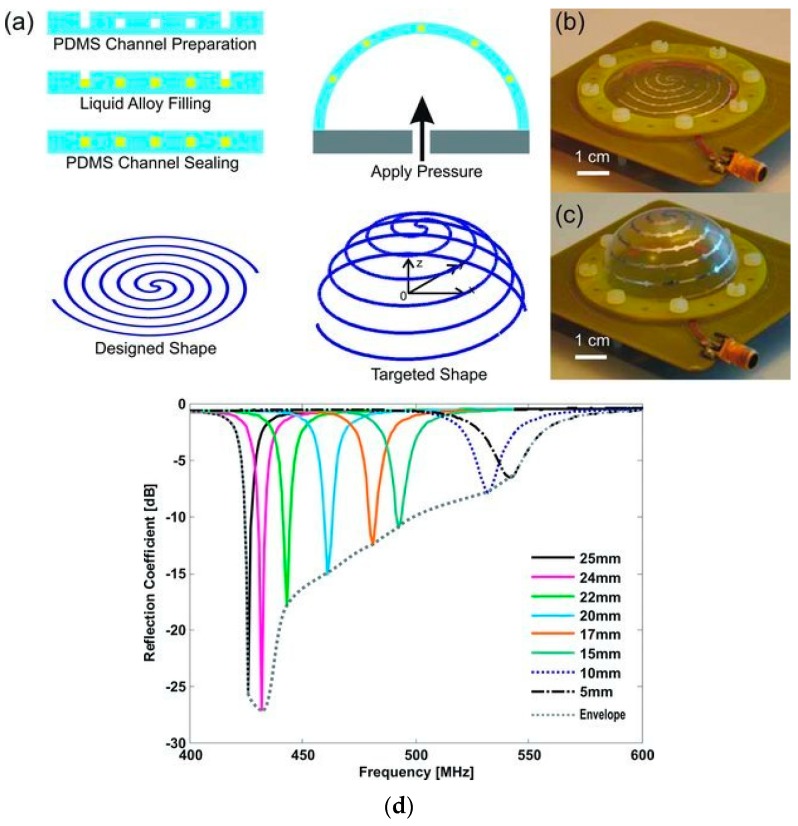
(**a**) Schematic illustrating ESA fabrication and operation; (**b**) implemented antenna prior and (**c**) after applied pressure; (**d**) variation in reflection co-efficient as the ESA’s dome height and corresponding frequency of operation changed [79]. Abbreviations: ESA; electrically small antenna.

Miniaturization is another antenna application where LM and associated patterning techniques can be utilized. Figure 14 depicts one such antenna in which a frequency tunable electrically small antenna (ESA) developed with Galinstan fluid within PDMS substrate encapsulation was presented [79]. In this antenna, frequency reconfigurability was achieved using a pneumatic pump, which varied the height of the dome structure to change the antenna’s operating frequency. In [79], the antenna’s frequency of operation could be varied from 426 MHz to 542 MHz by changing the height of the dome from 25 mm to 5 mm.

### 5.5. LM Antenna Arrays

Owing to the simplicity of fabricating simple antenna elements with LM, microfluidic channels used for simple patch, dipole, and loop antennas can be replicated in the form of antenna arrays. This method was used to achieve polarization reconfigurability in [64] using a superstrate. This superstrate consisted of a 4 × 4 microfluidic element array filled with EGaIn. The array elements were designed to achieve gains of 5.85 dBi and 5.86 dBi at 2.51 GHz for LHCP and RHCP, respectively. Similarly, frequency reconfigurability was achieved using 1 × 4 pixelated LM cells on both arms of a dipole antenna in [80]. Using continuous electrowetting technique, each pixel could be turned on and off with a switching time of between 30 and 90 milliseconds. Turning on a different number of the pixel (from one to four) on each dipole arm resulted in a resonant frequency variation from 2.51 GHz to 1.68 GHz, while maintaining an antenna efficiency greater than 70%.

**Figure 15:**
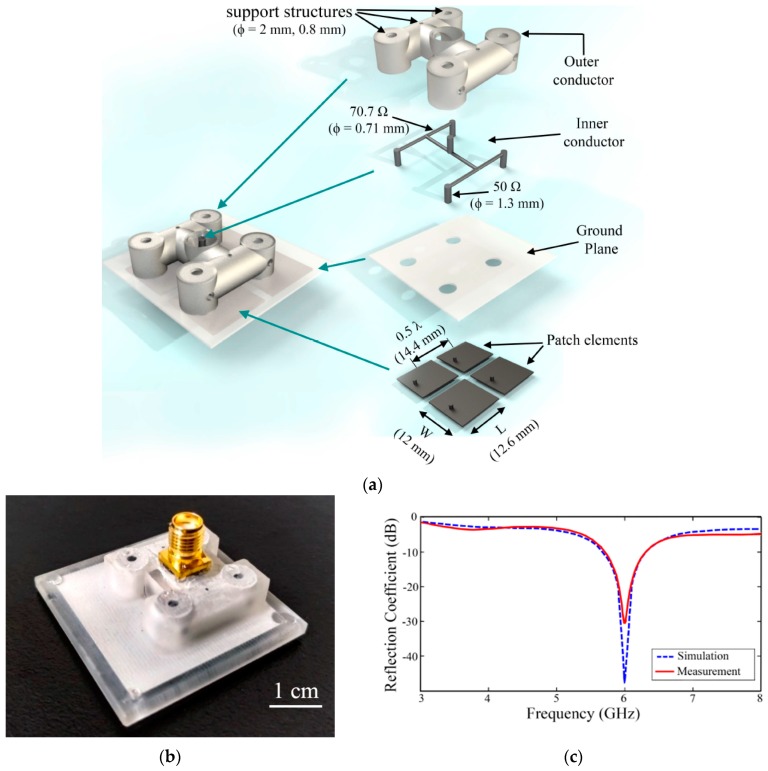
(**a**) Exploded view of the antenna array with 3D-printed Support structure and EGaIn patch elements; (**b**) fabricated antenna; (**c**) measured vs. simulated reflection coefficients as a function of frequency [52].

Compared to the standard antenna arrays with interconnects implemented in PCBs, [52] presented a unique method of developing the radiating elements and the corresponding interconnects in a 3D-printed structure, as shown in Figure 15a,b. The cavity developed in the 3D support structure were filled with EGaIn LM to form a 2 × 2 patch antenna array and tuned 50 Ω interconnects. Figure 15c shows that the measured resonant frequency, which matched simulations and operated at 6 GHz, which was developed using rapid prototyping technique. 

In summary, the LM antennas have diversified applications due to the controlled-flow property of conductive fluids. These applications include frequency, pattern, and polarization reconfigurable LM antennas. Here, tunabilty was achieved by controlled flow of the metal liquid, which is possible due to their low oxidization and stiction properties. LM antenna was also suitable for wearable application due to their extra ordinary elastic and deformable properties. This ensured that the LM antenna can be placed on parts of the human limbs like knee/arm joints, etc. Miniaturization is another antenna application where LM and associated patterning techniques can be utilized to have ESA conformal antenna with reasonable radiation characteristics

## 6. Discussion and Conclusions

This article reviews different aspects of liquid antennas including materials, fabrication methods, and their applications. Liquid metals, which include mercury, Galinstan, and EGaIn, exhibit long-term stability of electrical properties when encapsulated in microchannels [47], printed [26], or even sprayed [40] on commonly used flexible substrates like PDMS, EcoFlex, NinjaFlex, etc. LM fluids also possess outstanding elastic properties as radiative elements in antennas, where for example, 95% radiation efficiency was maintained for LM antenna despite a 50% applied strain [75]. Furthermore, negligible change in resistance of screen-printed LM pattern was observed after 1000 bending cycles in [35] as compared to an eight-fold increase in resistance of screen-printed copper pattern after 500 bending cycles as reported in [81]. Although copper-based antennas are highly efficient and can withstand moderate bending, they underwent irreversible plastic deformation when stretched by more than 2% [4]. When interfacing with traditional metal interconnects like Cu, Ag, and Al etc., the Ga in the LM alloys is known to cause corrosion and reduces long-term stability of antenna performance [24,36,37]. However, these issues can be addressed by Ni-plating the LM/metal pin interface or by treating it with 1-decylphosphonic acid, by HCl-vapor [36], or with PEDOT:PSS/GO composite [37]. Furthermore, the development of LM-based nanocomposites has not only resulted in cheaper and reliable fabrication processes but also increases in their electrical properties. For example, an addition of just 0.5% of SWNTs to EGaIn resulted in a 100% increase of its electrical conductivity [20].

Most common fabrication/patterning techniques for LM antennas include soft lithographic method, direct wiring technique, stencil printing, 3D printing, substrate milling, and pressure spraying. Among these LM patterning techniques, lithography is the most widely used method for developing rigid molds for flexible antenna development. These molds are primarily used to create microfluidic channels for flexible substrates to be subsequently filled with LM. Using this technique, functional LM antennas have been developed by injecting and pressure sintering LM nanoparticles in a 50 µm microchannel in PDMS, as reported in [27]. However, using a vacuum filling technique, a microchannel as thin as 5 µm developed in a PDMS substrate was successfully filled with LM [51] and may be replicated to develop and fill ultrathin patterns in LM antennas. 3D printing is an additive method used in antenna fabrication to print 3D structures hosting microfluidic channels using flexible and rigid substrates. It offers a quick and low-cost method to build complex fluidic channels as compared to the soft lithographic method. These 3D-printed structures generally have limited printing resolutions, for example, a microchannel width of 300 µm with VeroClear 3D-printed substrate material was reported in [38]. On the other hand, direct writing/printing of conductive traces is also possible by use of UV-assisted direct write technology and LM inks. Furthermore, direct 3D printing technology was used to develop a reconfigurable LM coil with state-of-the-art track width/spacing of 12 µm/50 µm in [54]. This 3D direct printing technique was reported as highly reliable and repeatable with an incredibly small track thickness of ∼2 µm, which can be exploited for next generation LM antennas. Pressure spraying LMs onto screens/masks to implement LM patterning onto substrates underneath is another prevalent antenna fabrication technique where a minimum track width/spacing of 50 µm/34 µm has been achieved after scrapping the excess LM left after spraying in [40].

Due to the integration of various wireless technologies/standards in the future 5G network, the requirement for adaptive antennas with frequency, polarization, and/or pattern diversities are highly desirable and are traditionally implemented using different types of switches. The predefined shape of switch-based reconfigurable antennas, limit their use for such applications. On the contrary, LMs require less biasing circuitry for tuning with enhanced power handling capability and have relatively simpler antenna structures. The amount of fluid in cavities can be controlled using micro pumps or electrochemically controlled capillary actions to physically change the size/position of radiating element and tune antenna’s frequency, polarization, and/or pattern. For example, continuous tuning in a wide frequency range from 0.66 GHz to 3.4 GHz was achieved by changing antenna length from 75 mm to 4 mm in [62]. Similarly, continuous polarization tuning from 0° to 180° was achieved using an LM-based parasitic circular patch antenna in [66] and continuous beam steering over 360° range was reported in [68].

Owing to flexibility and flow properties of LM-based fluids, and due to their reliable, robust, and cost-effective fabrication methods and tunability, LM-based antennas have found widespread use in applications like skin-attachable motion sensing systems, wireless power transfer, biomedical implantable devices, RFID tags [26], and recently in handheld cellular devices [78]. The flexibility and highly elastic nature of LM alloys enable development of biomedically compliant antennas to be placed on parts of the human limbs like knee/arm joints, etc. [75] or to be incorporated with tissue for comfortable wireless charging of implanted devices [77]. Here, the flexibility and RF power handling of the antenna allowed wireless charging of a discharged 120 mAh battery in just 18 minutes with 9 W of RF power at input of transmission coil. This maximum RF power handling capability of an LM antenna is nominally limited due to the temperature limitations of involved substrate or components like micropumps in [59]. The simplicity of fabricating simple antenna elements with LM, microfluidic channels used for simple patch, dipole, and loop antennas have also been replicated to form complex antenna arrays in [52].

To conclude, LM-based antennas might not represent a general alternative to standard high-frequency switching techniques used in modern communication devices because of their small electrical conductivities, slow switching speeds and micropump sizes. However, with recent development of better LM nanocomposites, fabrication/filling methods, and tuning/reconfigurability techniques, LM antennas do show a very promising prospect as part of the larger future 5G network, especially for applications demanding flexible antenna solutions.

## Figures and Tables

**Table 1:** Properties of popular conductive fluids used in antenna design. Additional LM material properties are available in [7,19,24]. (Silver nanoink and AgNW added for comparison only). Abbreviation: SWNT, single-walled carbon nanotube; AgNW, silver nanowire.

Liquid Material	Composition	Electrical Conductivity (10^6^ S/m)	Melting Temperature (°C)	Density @25 °C (g/cm^3^)
Mercury [2]	High purity Hg	1	−38.87	13.55
EGaIn [2]	75% Ga, 25% In	3.4	16	6.25
Galinstan [2]	68.5% Ga, 21.5% In, 10% Sn	3.4	−19	6.44
GaIn10 LM ink [25,26]	∼90% Ga, ∼10% In, 0.026 wt% O	2.9	16	∼6.05
EGaIn + 0.5% SWNT [20]	∼74.6% Ga, 24.9% In, 0.5 wt% SWNT	∼6.8	-	-
EGaIn nanoparticles [27]	EGaIn nanoparticles in PDMS substrate	0.092 (max)	-	-
Silver nanoink [28]	72 wt% Ag in organic solvent	∼20	-	-
AgNW [18]	AgNW in PDMS substrate	0.813	-	-

**Table 2:** LM Antenna patterning techniques based on soft/photolithography fabrication method. Abbreviations: LCP, liquid crystal polymer.

Patterning Technique	Antenna Type	Radiative/Substrate Material	Distinctive Features
LM injection in replica molded microfluidic channels [4]	Dipole	EGaIn/PDMS	150 µm thick microfluidic channels (1910–1990 MHz)
Multilayer non-meshed antenna structure with [29]	Microstrip patch	EGaIn/PDMS	Broad filled antenna area of ∼16 mm^2^ supported by PDMS post structures (3.45 GHz)
Selective wetting of LM patterns [43]	RF Coil	Galinstan/PDMS	50 µm line width, power and data transfer (∼14 MHz)
PDMS microchannel glued to LCP substrate and LM flow controlled by micro pump [23]	Monopole	Mercury/(PDMS+LCP+RT 5880)	500 µm wide and 250 µm thick channel. Technique not viable with Ga alloys. Needs micropumps. (∼1.3–5.2 GHz)
LM cavity between replica molded Ecoflex attached to PET sheet with Cu tape layer [45]	Patch	EGaIn/(EcoFlex+PET+Cu Tape)	Mechanical bonding/sealing may get damaged after LM injection cycles (2.45 GHz)
Pressure sintering of LM nanoparticles after micro-channel injection [27]	Dipole	(EGaIn nanoparticles)/PDMS	50 µm thick channel. Three times lower pressure for inserting LM into microchannel (∼2–3 GHz)
Vacuum filling of patterned microchannels [51]	RF Coil	EGaIn/ PDMS	Capable of filling as small as ∼5 µm wide microchannel. Very fast process. Also suitable for highly complex/branched channels.

**Table 3:** LM Antenna patterning techniques based on printing/direct writing fabrication methods. Abbreviations: IFA, inverted-F antenna.

Patterning Technique	Antenna Type	Radiative/Substrate Material	Distinctive Features
LM injection into 3D-printed channel [31]	IFA	Galinstan/NinjaFlex	0.7 mm channel diameter. Highly Flexible and miniature design (885 MHz)
LM injection in to 3D-printed microchannels using micropumps [38]	PASS	(EGaIn+ mineral oil)/VeroClear	300 µm microchannel width. Micropumps were needed (∼2.1–3.5 GHz)
Vacuum filling of rigid 3D-printed substrate [39,52]	Planar & Helical [39], Patch Array [52]	EGaIn/ (M3 crystal)	500 µm cavity diameter (Up to 8 GHz) [39], Complex 3D array support structures (6 GHz) [52]
LM composite filled in UV-cured direct written epoxy/SWNT boundary [20]	Patch	(EGaIN +SWNT)/PDMS	Less than 4.3% return loss fluctuation even after 12 months of storage at room temperature (4 GHz)
2D Direct printing [26]	RFID Coil	GaIn10 ink/ plastic	0.2 mm/0.5 mm line width/spacing. Compatible with paper, plastic, latex, and textile substrates. Low cost.
3D Direct printing LM tracks [54]	RF Coil	EGaIn/glass	12 µm/50 µm track width/spacing (minimum track width of ∼2 µm). Printed 2D and 3D tracks with high reliability and repeatability (∼7.2 GHz)

**Table 4:** LM Antenna patterning techniques based on fabrication methods involving LM spraying.

Patterning Technique	Antenna Type	Radiative/Substrate Material	Distinctive Features
Tape transfer of patterns to half cured PDMS and subsequent LM spraying and encapsulation [44]	RF Coil	Liquid alloy/PDMS	Trace thickness/spacing of 200 µm/500 µm. Reliable and uniform LM deposition in a trace (140 kHz)
LM sprayed onto substrate covered by polyamide mask using pressurized air gun [40]	Patch	Galinstan/ RT5880	Minimum line thickness/spacing of 50 µm/34 µm using scrapping of sprayed LM using needle (3.8 GHz)
Pressurized LM spray onto polyester screen mesh [35]	RFID	Galinstan/ PVC	∼250 µm thick tracks. Compatible with substrates having rough surfaces like paper, plastic, PDMS and glass (986.7 MHz)
